# 

**Published:** 1875-03

**Authors:** 


### Books and Pamphlets Received.

Cyclopædia of the Practice of Medicine. Edited by Dr. H. von Ziemssen. Vol. II. Acute Infectious Diseases. American translation edited by Albert H. Buck, M. D. New York: Wm. Wood & Co., 1875.

Lectures on Diseases of the Respiratory Organs, Heart and Kidneys. By Alfred L. Loomis, M. D., Prof. of Pathology, etc., Med. Dept. University of New York. New York: Wm. Wood & Co., 1875. Buffalo: H. H. Otis.

Syphilitic Lesions of the Osseous System in Infants and Young Children. By R. W. Taylor, M. D. New York: Wm. Wood & Co., 1875. Buffalo: H. H. Otis.

On Functional Derangements of the Liver. Being the Croonian Lectures delivered at the Royal College of Physicians in March, 1874. By Charles Murchison, M. D., LL. D., F. R. S. New York: Wm. Wood & Co., 1875. Buffalo: H. H. Otis.

THE BUFFALO

**Medical and Surgical Journal**

PUBLISHED MONTHLY,

Containing Original Articles, Reports of Medical Societies and Hospitals, Editorial, Reviews, Correspondence, Army News, etc., etc ; including the usual variety of Medical Periodical Publications. Specimen copies sent on application.

Address Buffalo Medical & Surgical Journal, Buffalo, N. Y.

TERMS OF SUBSCRIPTION, - - $3.00 PER YEAR, IN ADVANCE.

